# Y-Chromosome Analysis in Retuertas Horses

**DOI:** 10.1371/journal.pone.0064985

**Published:** 2013-05-31

**Authors:** Claudia Brandariz-Fontes, Jennifer A. Leonard, José Luis Vega-Pla, Niclas Backström, Gabriella Lindgren, Sebastian Lippold, Ciro Rico

**Affiliations:** 1 Estación Biológica de Doñana (EBD-CSIC), Seville, Spain; 2 Facultad de Medicina Veterinaria, Universidad de Panamá, Panama City, Panama; 3 Conservation and Evolutionary Genetics Group, Estación Biológica de Doñana (EBD-CSIC), Seville, Spain; 4 Laboratorio de Investigación Aplicada, Cría Caballar de las Fuerzas Armadas, Córdoba, Spain; 5 Department of Evolutionary Biology, Uppsala University, Uppsala, Sweden; 6 Department of Animal Breeding and Genetics, Swedish University of Agricultural Sciences, Uppsala, Sweden; 7 Department of Evolutionary Genetics, Max Planck Institute for Evolutionary Anthropology, Leipzig, Germany; University of York, United Kingdom

## Abstract

Several studies based on a variety of genetic markers have attempted to establish the origins of horse domestication. Thus far a discrepancy between the results of mitochondrial DNA analysis, which show high levels of diversity, and results from the Y-chromosome, with almost no genetic variability, has been identified. Most previous work on the horse Y-chromosome has focused on widespread, popular breeds or local Asian breeds. It is possible that these breeds represent a reduced set of the genetic variation present in the species. Additional genetic variation may be present in local breeds and ancient feral populations, such as the Retuertas horse in Spain. In this study we analyzed the Y-chromosome of the Retuertas horse, a feral horse population on the Iberian Peninsula that is at least several hundred years old, and whose genetic diversity and morphology suggests that it has been reproductively isolated for a long time. Data from the Retuertas horse was compared to another 11 breeds from the region (Portugal, Spain and France) or likely of Iberian origin, and then to data from 15 more breeds from around the globe. We sequenced 31 introns, Zinc finger Y-chromosomal protein (*ZFY*) and anonymous Y-linked fragments and genotyped 6 microsatellite loci found on the Y-chromosome. We found no sequence variation among all individuals and all breeds studied. However, fifteen differences were discovered between our data set and reference sequences in GenBank. We show that these likely represent errors within the deposited sequences, and suggest that they should not be used as comparative data for future projects.

## Introduction

In stark contrast to the high levels of maternally inherited mitochondrial DNA variation observed in domestic horses, previous studies of the paternally inherited Y-chromosome have reported a deficiency of genetic variation among a wide range of domestic horses including both warm- and coldblooded breeds [1]–[8]. This observed lack of diversity is unexpected given that most mammals, including other domestic species such as cattle, sheep and dog [9]–[13] have at least some variation on the Y-chromosome and research using homologous Y-chromosome markers has found them to be variable in other equids [5], [6]. Further, a recent study revealed extensive genetic variability in Y-chromosome sequences from pre- and early domesticated horses, demonstrating that Y-linked genetic diversity was present in the wild ancestor and in past populations of domesticated horses [14].

Even if variation was present in early domesticated horses, it could have been lost quickly through drift within breeds due to low effective population size as a consequence of breed formation, especially in males where inter-individual variance in reproductive output is immense. However, drift should have acted independently within each isolated breed and it is therefore expected that different alleles of polymorphisms that were present in the progenitor population should be preserved or fixed in different isolated populations. The only observations of genetic variation reported in the modern domestic horse Y-chromosome is in a study of microsatellite allelic diversity of several local breeds from China [15] which suggests that Y-chromosome polymorphisms may be more likely to be found in local breeds or in ancient feral populations that have not contributed to the formation of the major popular breeds around the world. In support of that view, Iberian horse breeds show high levels of overall genetic variation as compared to other European horse populations [16]. For these reasons, we hypothesize that the Retuertas horse, a feral population living in the Doñana National Park in southern Spain, may harbor Y-chromosome polymorphisms not previously observed in other horse breeds. This feral population has been reproductively isolated from other breeds for substantial time and does not cluster with either of the two major clades of European and North African breeds based on microsatellites and allozyme data [17]. Here we scanned for Y-linked genetic polymorphisms in Retuertas horse using 31 previously reported Y-chromosome specific loci and we also genotyped six microsatellites located on the horse Y-chromosome.

## Materials and Methods

### Study System

The Retuertas horse is a feral population that lives in the Guadalquivir marshes in southern Spain, including Doñana National Park, and has been shown to be genetically differentiated from 10 other Iberian, North African and European breeds using 22 microsatellite and 5 allozyme loci [17]. Their morphological and physiological features are distinct from Spanish Purebred, Arabian and other Iberian horses of Celtic origin (e.g. the average height is only 1.42 m and its adaptable to a hostile marsh environment). Furthermore, a private allele of a major gene involved in lipid metabolism (Carboxilesterase) found in a systematic protein electrophoresis analysis, suggested a long-term genetic isolation of the Retuertas horse population [17]. This suggests that this population has remained largely reproductively isolated from other domestic breeds and likely represents a population of horses that has survived in the Guadalquivir marshes in southern Spain for several hundred years [18]. Extensive field observations and information on management of the herd, especially males, was used to construct a likely pedigree of all individuals in the park.

### Ethics Statement

Animal Care and Use Committee approval was not obtained for this study because no animals were handled specifically for this experiment. Blood samples were collected by qualified veterinarians through their routine practice, in the framework of official programs aimed at the identification, health control and parentage confirmation of the breeds and populations included in our study. Therefore, the legal restrictions defined in “Spanish Law 32/2007 of November 7, on the care of animals in their husbandry, transportation, testing and sacrifice” do not apply, as they are waved in the case of non-experimental procedures and routine veterinary practices with livestock species, in Article 3d of the above-mentioned Law.

### Samples and DNA Extraction

Total genomic DNA was extracted from frozen blood samples collected for reasons other than this project using QIAmp DNA Blood Mini Kit (Qiagen), according to the manufacturer’s instructions.

We investigated 46 male horses from 12 different breeds currently present in the region (Portugal, Spain and France) or thought to have Iberian ancestry: Retuertas, Spanish Purebred (also called Andalusian Horse), Asturcon, Breton, Losino, Mallorquin, Menorquin, Pottok, Uruguayan Creole, Lusitano, Marismeño and Lipizzan (Table 1). DNA from two Retuertas females was used as a control. Given the pedigree, the six Retuertas males were selected to represent all paternal lineages present in the population. In the second step of the analyses the dataset was expanded with data from previously published reports [7], [14].

**Table 1:** Breeds analyzed in this study.

Breed	Y-chrom loci*	Microsatellites	Geographic region
*Retuertas*	6	6	Doñana National Park, Spain
SpanishPurebred	5	4	Córdoba-Andalusia, Spain
*Asturcon*	4	4	Asturias, Spain
*Breton*	3		France
*Losino*	4	3	Burgos, Spain
*Mallorquin*	4	3	Mallorca
*Menorquin*	4	3	Menorca
*Pottok*	4	3	Basque Country, Spain
*Uruguayan* *Creole*	2		Uruguay
*Lusitano*	3		Portugal
*Marismeño*	5	4	Huelva, Spain
Lipizzan	2		Slovenia

Breed name, sample size per analysis, and geographic region of the stallions from the 12 horse breeds from which new data has been generated here. Breeds from which no data was previously available are indicated in italics.

* Y-chrom loci are the six fragments with putative SNPs: *Amely6, SRY, Eca-Y2B17,Eca-Y3B1, Eca-Y3B12, Eca-Y3B19.*

### PCR Amplification and Sequencing of Y-specific Fragments

Initially we sequenced 31 Y-chromosome introns, ZFY and anonymous Y-linked fragments reported in [7] from 6 Retuertas stallions (Table 2). These 31 loci cover more than 12.3 kb in total and were amplified through polymerase chain reaction (PCR) and then directly sequenced. DNA from two females was included as a negative control in the PCRs to verify Y-chromosome specific amplifications. Six of the 31 fragments yielded an apparently different sequence in the Retuertas horse from the reference sequences in GenBank: the two Y-chromosome introns *AMELY 6* [9] and *SRY* [20], and the four anonymous Y-linked fragments *Eca-Y2B17, Eca-Y3B1, Eca-Y3B12* and *Eca-Y3B19* [5].

**Table 2:** Description of horse Y-chromosome nuclear markers.

Locus	L		T_m_	Forward Primer (5′–3′)	Reverse Primer (5′–3′)		Acc. No.
*AMELY1*	394		TD	ACATGTTTTTCATTCAGAAATAT	GTTATTGAGGTACTTAAAGTGT	AB091794	
*AMELY2*	358		TD	TTTACTACTTTGAAAAACACTTT	ATTGGATTTTAGGGGTTCTT	AB091794	
*AMELY3*	481		TD	CCCTAAAATCCAATAGGGTT	CATGTATGTAATTAGTCCTT	AB091794	
*AMELY4*	488		TD	CTATTTCACAAGCTTGAATGC	TCACCACATACAAGTCATAAG	AB091794	
*AMELY6*	470		TD	CTTCACGTTCAAATGTGTGAC	TCATTACAGATCACAACATGG	AB091794	
*AMELY7*	215		TD	CATGTTGTGATCTGTAATGAA	AATTAAATGACTTTCTCAGGG	AB091794	
*AMELY8*	478		58	CAAGATGTTTTTCCATTCCTC	TTTTGAAGTGTGGGCATTAAT	AB091794	
*AMELY9*	203		56	ATCTGTAGAAGGGAATTAATG	GAAACTGTGAAAGAGGAATAG	AB091794	
*AMELY11*	245		58	CTCTGAAGTGGTACCAGAACA	ATTGGCTCCATTGACTCTCTG	AB091794	
*SMCY3*	848		56	ATTTACCCTTATGAAATRTTT	TCAAATGGGTGWGTGTACAT	AY532887	
*SMCY7*	341		60	TGGAGGTGCCCRAARTGT	AACTCTGCAAASTRTACTCCT	AY532888	
*SRY*	452		TD	CGGACTTTCTCACGGTGATT	CAAGACTGGTTTCTCACAGC	AB004572	
*ZFYG*	539		60	CCGAAATTGCTGATGAGGTT	TATGTGCAAGAGGGCACTTG	AY532846	
*ZFYH*	579		56	TCTGAACCGGCGAAATGT	TCATCCTACCCAAAGCCAAC	AY532847	
*ZFY27A*	323		58	CTAACTAAAGTTTTCAGTTTTG	AAGATGGAGATATTGCTCTA	AY532848	
*ZFY43A*	435		60	GAAATAAACCCACACATACTCT	TATATGCGTGATGCTTGC	AY532849	
*ZFY44A*	391		56	TGGTTTTGGTGTATTACATC	ATGAAAGGATAAACAAAATG	AY532850	
*ZFY46A*	341		60	TGCACATTTCCTTTAATCT	GCACATTAAAGAGAAACCTT	AY532851	
*ZFY50A*	252		60	TTAAAAAAGACCTTCTCCTA	CCTTTAGCTTTTGTTTTCT	AY532852	
*ZFY50B*	314		60	AAAAGTTATTGTCAGCTTCAGC	TTCTGCCCTTTTCCTCTTC	AY532853	
*ZFY51A*	353		60	CCAGGGAGACAGTGAAAGTAGG	GGTAGGGCACCTTGACTACACT	AY532854	
*ZFY52A*	381		56	ATCCTTTCTTTTATTCCTTT	CATGCAAACTTAACCACTT	AY532855	
*ZFY53A*	414		56	TTTCCTTTCAGTTACCTTTCAT	CTACCTGTTGATGGGATTGA	AY532856	
*ZFY53B*	358		60	AAGGTAAAGTGTCTGTTCCA	AGACTCTCTCAGGAAAACTTAT	AY532857	
*ZFY55A*	342		60	CTGTTTAGCAAATAATTGTT	TAGGCTATATCATGCAGAT	AY532858	
*ZFY55B*	426		58	ATTAGGTGATTGCCTGAT	CAAAACTAGGGAGACAGTAA	AY532859	
*Eca-Y2B17*		438	TD	TTCAGTCCTGCTTTCTCCTCA	CAGGATGTGCCATGTGATTG	G72335	
*Eca-Y3B1*		468	TD	TGGGTTAATGGGATTTGGTG	CAAGCACAGCTCTGTATCAA	G72336	
*Eca-Y3B8*		445	TD	CCCAAGTTCCTTGCCATC	AAATTGAAGAGGCCCCAAAG	G72337	
*Eca-Y3B12*		392	TD	GGGAGGCACTGGAAAGTACA	GGTGGAGGAATCAGCTGGAG	G72338	
*Eca-Y3B19*		215	TD	AAGCCTTTCATGGAAATTGG	TTACGCAGACATCCTGGACA	G72339	

These loci all come from Lindgren et al. [7]. The first 12 loci are Y chromosome introns anf have the prefixes *AMELY*, *SMC* and *SRY*. The next 14 loci are based on subclones from a *ZFY*-positive BAC clone and have the prefix *ZFY*. The last five loci are anonymous Y-lined fragments and they have the prefix *Eca-Y*. Primers for each locus, along with annealing temperature (T_m_), length of amplicon (L) and GenBank accession numbers (Acc. No.) are organized by locus type. The introns, *ZFY* and anonymous Y-linked fragments were sequenced with amplification primers.

In the second stage, we amplified and Sanger sequenced these six fragments covering 2.5 kb from an additional 40 stallions representing 11 additional breeds in order to determine if the apparently new Y-chromosome variant was also present in other regional or related breeds. Ten of these breeds have no published Y chromosome sequences (Table 1). We included 5 stallions from two previously sequenced breeds (Spanish Purebred and Lippizan) to verify our findings with those reported in GenBank. Two female samples were again included as negative PCR controls.

The PCR amplification of Y-chromosome fragments was performed in 15 µl reactions containing 25–50 ng of template DNA, 2.5 U BioTaq DNA Polymerase (Bioline), 0.2 mM each of the four deoxynucleotide triphosphates (dNTPs), 30 ng BSA (Sigma-Aldrich), 50 mM KCl, 2 mM MgCl_2_, 10 mM TrisHCl (pH 8.3), and 15–25 pmoles of each primer (Table 2). Amplification reactions were performed in a MJ Research thermocycler, Model PTC-100. The PCR thermal cycling conditions consisted of an initial denaturation at 95°C for 3 minutes (min), followed by 35 cycles of 95°C denaturation for 30 seconds (sec), annealing for 30 sec at 56–60°C (annealing temperatures for each primer detailed in Table 2) and extension at 72°C for 60 sec, followed by a final extension at 72°C for 3 min. Alternatively, a touchdown PCR profile was used, where the annealing temperature ranged from 55–45°C with a decrease of 0.5°C/cycle for 20 cycles, followed by a constant annealing temperature of 45°C for 10 cycles. All reactions, including the female controls and reaction negative controls, were checked on 1.5% agarose gels stained with CyberSafe dye (Invitrogen).

All primers were confirmed to amplify Y-chromosome-specific products by using female horse DNA samples as controls. Successful amplifications were purified using the enzymes exonuclease I and shrimp alkaline phosphatase (USB Corporation,) and Sanger sequenced with BigDye chemistry (Applied Biosystems) in both directions using the primers used for amplification. Nucleotide sequences were determined on an ABI PRISM 3130×1 automated sequencer (Applied Biosystems).

To verify the results approximately 50% of the samples were replicated in a second PCR, with the same conditions. These replications were also sequenced in both directions using the same primers used for amplification by the commercial service offered by Macrogen (Macrogen, Korea).

### Sequence Analysis

Sequences were edited, assembled and aligned using the program Sequencher (Gene Codes Corporation). Sequences generated here were compared to all sequences in GenBank with the Basic Local Alignment Search Tool (BLAST) (http://blast.ncbi.nlm.nih.gov) [21]. Reference sequences from the six loci with putative SNPs were downloaded from GenBank (Table 3). Homologous sequences from all equid species for which they were available were also obtained (Table S1). In addition to the reference sequences, two other domestic horse *SRY* sequences not associated with any publication are in GenBank (accession numbers AC215855 and HM103387). Sequences from an ancient domestic horse were available for all six loci [14]. Additionally, the original sequences from Lindgren et al. [7] and alignment files from Lippold et al. [14] including sequences from Wallner et al. [22] that had not been deposited into GenBank were obtained and all sequences for each locus were aligned and compared in BioEdit (Ibis Biosciences, http://www.mbio.ncsu.edu/BioEdit/bioedit) [23].

**Table 3:** Putative polymorphic positions identified in the Y-chromosome.

Locus	Acc. No.	Position	Polymorphism	GenBank	This study
*AMELY 6*	AB091794	2757	Substitution	G	A
		2854	Substitution	C	T
		2881	Indel	A	–
*SRY*	AB004572	825	Substitution	T	G
		826	Substitution	T	G
		1033	Indel	–	G
*Eca-Y2B17*	G72335	173	Substitution	T	C
		460	Substitution	A	G
*Eca-Y3B1*	G72336	201	Substitution	G	A
		265	Substitution	C	A
		421	Substitution	G	A
*Eca-Y3B12*	G72338	49	Substitution	G	A
		281	Substitution	C	T
*Eca-Y3B19*	G72339	115	Substitution	T	A
		190	Substitution	G	A

Differences observed when comparing the sequences available in GenBank to the sequences we generated here and the ones generated by Lindgren et al. [7] (in total 150 stallions from 25 different horse breeds). The first column lists the name of the locus, followed by the GenBank accession number (Acc. No.) and the position in the GenBank reference serquence of the mismatched base pairs. Polymorphism refers to the nature of the difference- both single nucleotide sequence differences (substitution) and length differences (indel) were identified, and that is followed by the state of that base pair in the reference sequence (GenBank) and that identified here (this study).

### PCR Amplification and Genotyping of Y-chromosome Specific Microsatellite Markers

We also screened for length variation in six Y-chromosome-specific microsatellite loci [6] in 30 male horses from eight breeds (Tables 1,4,5), including the Retuertas horse. The loci screened are: *Eca.YA16, Eca.YH12, Eca.YM2, Eca.YP9, Eca.YE1,* and *Eca.YJ10* [6]. DNA of two female horses was used as a control in all PCR reactions. Typing of microsatellite loci was carried out with tailed primers that were used to add a phosphorescent dye in a second stage of amplification as previously described [24], [25].

**Table 4:** Description of horse Y-chromosome specific microsatellite markers.

Locus	AS	T_m_	Dye	Forward Primer (5′–3′)	Reverse Primer (5′–3′)	Acc. No.
*Eca.YH12*	102	58	FAM	CGAACAGGTGACGAAGCATC	GCAGACATGCACACCAACC	BV005747
*Eca.YM2*	117	54	PET	TGGTTCAGATGGTGTATTTTGTT	TTTGCAGCCAGTACCTACCTT	BV005725
*Eca.YP9*	213	54	PET	AAGCACTGCCTTTTGGAATC	AACCCTGGACTTTCTTTTGAA	BV005726
*Eca.YE1*	196	60	NED	CTTCACTCCCGACCAAGAGA	GTGTGTCGTGCCGTGTTTAC	BV005726
*Eca.YJ10*	213	60	FAM	AGTTCCCCTGCACACCT	TGCCTCCCACAGCCATAC	BV005728
*Eca.YA16*	156	60	VZC	TGACTGGAAATTGAAGATG	TTGTAGCAACAAAGTAACAC	BV005729

These loci come from Wallner et al. [6]. Primers for each locus, along with allele size (AS), annealing temperature (T_m_), dye label (Dye) and GenBank accession numbers (Acc. No.).

**Table 5:** Y chromosome microsatellite loci.

Locus	Repeat motif	Ni	Nb	*Ec*	*Ep*	*Ea*	Acc. No.
*Eca.YH12*	(GT)_13_ 1	1057	77	96^1,2^	100^1^	106^3^	BV005747
				100^1^	102^1,3^		
				102^1,3,4^			
*Eca.YM2*	(CA)_12_ 1	1057	77	116^1,4^	116^1^	110^1^	BV005725
				117^3^	117^3^	111^3^	
				119^2^		112^1^	
*Eca.YP9*	(CA)_10_ TAT(CA)_6_ 1	1057	77	213^3^	213^3^	196^3^	BV005726
	(CA)_10_ TA(CA)_6_ 5			214^1^	214^1^	197^1^	
				215^2^			
				218^4^			
*Eca.YE1*	(CA)_10_ 1	1057	77	196^1,3^	196^1,3^	191^1^	BV005726
	(CA)_10_CT(CA)_6_ 5			199^2,4^		192^3^	
*Eca.YJ10* 6	(CA)_3_ CG (CA)_6_ 1	1027	69	212^4^	213^1,3^	213^3^	BV005728
				213^1,3^			
*Eca.YA16*	(TG)_3_ TA (TG)_18_ 3	1057	77	152^3^	158^3^	156^3^	BV005729
	(TG)_3_ TA (TG)_16_ 3			156^2,3^	159^1^		
	(GT)_3_ TAT (GT)_19_ 1			157^1^	161^1^		
	(TG)_3_ TA (TG)_19_ 5						

1 Reference [6].

2 This study.

3 Reference [15].

4 Reference [8].

5 From GenBank reference (Acc. No) sequence listed in last column.

6 Found to amplify in female horses under some conditions.

Nomenclature of loci follows Wallner et al. [6]. The motif was sequenced only in horse, and then genotyped in horse (*Ec*), Przwalski’s horse (*Ep*) and donkey (*Ea*) samples independently in a subset of the studies as indicated. The number of individuals typed (Ni) and number of breeds types (Nb) is a compilation of data only for horse only from [6], [8], [15] and this study. Allele sizes reported in the different studies for the different species are listed. Importantly, the same allele may be scored differently by different individuals, when run on different machines, or between different runs on the same machine. Hence, evidence for polymorphism can only be deduced in cases when multiple alleles have been reported in the same study.

PCR amplifications were performed in 15 µl reactions each containing 20–30 ng DNA, 5 pmol of specific forward primer with its M13 tail, 20 pmol of the FAM labeled universal M13 primer, 20 pmol of specific reverse primer, 1× magnesium-free PCR buffer with KCl, 2.5 mM MgCl_2_, 1.5 µg BSA, 2 mM dNTPs, and 1.25 U BioTaq DNA Polymerase (Bioline). Amplifications were carried out using a MJ Research thermocycler, Model PTC-100. Cycling conditions were done in two stages without opening the tubes in between. As the M13 primer requires a 53°C annealing temperature [25], we added eight cycles at the end of the PCR cycles to incorporate the M13 fluorescently labeled primer with the previously formed amplicons. Amplification reactions were: 94°C for 1 min, then 25 cycles of 94°C for 30 sec, 54–60°C (Table 4) for 30 sec and 72°C for 30 sec. This was followed by 8 cycles to incorporate the dye-labeled M13 consisting of: 94°C for 30 sec, 53°C for 30 sec and 72°C for 30 sec, and a final extension at 72°C for 10 min. To confirm amplification, 5 µL of each product was electrophoresed on a 2% agarose gel at 100v for 45 min and visualized with CyberSafe dye (Invitrogen). Size of each PCR product was determined using an ABI 3130 DNA Sequencer (Applied Biosystems) with GeneScan LIZ 500 internal size standards (Applied Biosystems). Fragment size analysis was performed using GeneMapper V3.7 software (Applied Biosystems).

## Results

### Y-chromosome Fragments

PCR was successful for all six male Retuertas horses at all 31 Y-chromosome non-microsatellite loci and no amplicons were found in female controls. These sequences were completely monomorphic in the six male individuals sequenced. Twenty-five Y-chromosome fragments, totaling 9.9 kb, were identical to previously published sequences [5], [19], [20], available in GenBank (Table 2). A total 15 single nucleotide differences were identified when we compared our other six sequences to the sequences from GenBank (Table 3, Table S1). These were initially considered putative single nucleotide polymorphisms (SNPs) within the Retuertas breed. To confirm this unexpectedly high level of divergence, we sequenced these six putatively polymorphic fragments, in total 2.5 kb, in 40 additional male horses originating from 11 breeds: Spanish Purebred, Asturcon, Breton, Losino, Mallorquin, Menorquin, Pottok, Uruguayan Creole, Lusitano, Marismeño and Lipizzan. This included two breeds that had already been sequenced at these loci in another study, the Spanish Purebred and Lippizan [5]. All PCRs yielded a single amplicon from the male samples and none from the female controls verifying Y-linkage. All of the additional 40 male horses sequenced at these six fragments were identical, both to each other and to the six male Retuertas horses, and thus yielded the same 15 differences in comparison to the GenBank sequences (Table S1). These sequences have previously been reported in the literature from many individuals from a wide variety of breeds (Table 6) and are now deposited in GenBank (JX888707–JX888713).

**Table 6:** Breeds that have been studied for the six putatively polymorphic Y chromosome loci.

Breed	*Amely6*	*SRY*	*Y2B17*	*Y3B1*	*Y3B12*	*Y3B19*	Total
Ardennais	4^1^	4^1^	4^1^	4^1^	4^1^	4^1^	4
Akhal Teké	1^2^+2^1^	1^2^+2^1^	1^2^+2^1^	1^2^+2^1^	1^2^+2^1^	1^2^+2^1^	3
Andalusian	5+1^2^	5+1^2^	5+1^2^	5+1^2^	5+1^2^	5+1^2^	6
Appaloosa			1^2^			1^2^	1
Arabian	1^2^+4^1^	1^2^+4^1^	1^2^+4^1^	1^2^+4^1^	1^2^+4^1^	1^2^+4^1^	5
Asturcon	4	4	4	4	4	4	4
Austrian Warmblood	1^2^	1^2^	1^2^	1^2^	1^2^	1^2^	1
Barb			1^2^			1^2^	1
Breton	3	3	3	3	3	3	3
Caspian Pony	3^1^	3^1^	3^1^	3^1^	3^1^	3^1^	3
Connemara	4^1^	4^1^	1^2^+4^1^	4^1^	4^1^	1^2^+4^1^	5
Exmoor	4^1^	4^1^	4^1^	4^1^	4^1^	4^1^	4
Fjord	4^1^	4^1^	4^1^	4^1^	4^1^	4^1^	4
Gotland	4^1^	4^1^	4^1^	4^1^	4^1^	4^1^	4
Icelandic Horse	1^2^+4^1^	1^2^+4^1^	1^2^+4^1^	1^2^+4^1^	1^2^+4^1^	1^2^+4^1^	5
Khuzestan Arab	3^1^	3^1^	3^1^	3^1^	3^1^	3^1^	3
Kladruber	1^2^	1^2^	1^2^	1^2^	1^2^	1^2^	1
Lipizzaner	2+8^2^	2+8^2^	2+8^2^	2+8^2^	2+8^2^	2+8^2^	10
Losino	4	4	4	4	4	4	4
Lusitano	3	3	3	3	3	3	3
Mallorquin	4	4	4	4	4	4	4
Malwari	2^1^	2^1^	2^1^	2^1^	2^1^	2^1^	2
Mangalarga Marchador			1^2^			1^2^	1
Marismeño	5	5	5	5	5	5	5
Menorquin	4	4	4	4	4	4	4
Miniature			1^2^			1^2^	1
Mongolian native horse	1^2^	1^2^	1^2^	1^2^	1^2^	1^2^	1
New Forest Pony			1^2^			1^2^	1
Noric	1^2^	1^2^	1^2^	1^2^	1^2^	1^2^	1
North-Swedish	4^1^	4^1^	4^1^	4^1^	4^1^	4^1^	4
Norwegian Fjord			1^2^			1^2^	1
Old Wuerttemberger			1^2^			1^2^	1
Oldenburger			1^2^			1^2^	1
Paint			1^2^			1^2^	1
Pinto			1^2^			1^2^	1
Pottok	4	4	4	4	4	4	4
Quarter Horse	1^2^	1^2^	1^2^	1^2^	1^2^	1^2^	1
Retuertas	6	6	6	6	6	6	6
Saddlebred			1^2^			1^2^	1
Shagya Arabian Shire			1^2^			1^2^	1
Shetland	4^1^	4^1^	4^1^	4^1^	4^1^	4^1^	4
Shetland Pony	1^2^	1^2^	1^2^	1^2^	1^2^	1^2^	1
Tarpan-like horse	1^2^	1^2^	1^2^	1^2^	1^2^	1^2^	1
Thai Pony	2^1^	2^1^	2^1^	2^1^	2^1^	2^1^	2
Thoroughbred	1^2^+4^1^	1^2^+4^1^+1^4^	1^2^+4^1^	1^2^+4^1^	1^2^+4^1^	1^2^+4^1^	6
Tinker			1^2^			1^2^	1
Trakehner	1^2^	1^2^	1^2^	1^2^	1^2^	1^2^	1
Trotter			1^2^			1^2^	1
Uruguayan Creole	2	2	2	2	2	2	2
Other/unidentified	1^3^						1
**Total**	119	120	134	119	119	134	

1 Reference [7].

2 Reference [5].

3 Reference [19].

4 Reference [20].

List of domestic horse breeds represented in the literature and sequenced here for the six Y chromosome loci showing differences in sequence between studies. The original sequences from Lindgren et al. [7] are 100% identical to the sequences presented in this study.

### Y-chromosome Microsatellite Markers

All the samples were visually inspected on an agarose gel before being run on the sequencer to verify the correct amplification of the six equine Y-chromosome specific microsatellites. Some loci were sensitive to PCR conditions. Locus *Eca.YH12* yielded multiple amplicons of unexpected size and locus *Eca.YJ10* amplified in the female samples at some annealing temperatures (56–57°C). For this reason locus *Eca.YJ10* was discarded from further analyses. The five microsatellites which passed the verification process yielded no variation across all 32 male samples from 8 breeds. This set of markers includes *Eca.YA16*, a locus at which variation was reported in a recent study of microsatellite allelic diversity in Chinese domestic horse breeds [15].

## Discussion

### Sequence Variation

Consistent with results from previous studies, complete monomorphism of Y-chromosome sequences generated for this study was identified. All individuals sequenced here matched 100% to the sequences generated in another lab for a separate study [7]. The only report of Y-linked sequence variation within a domestic horse is from an ancient individual [14]. However, both standing genetic variation and fixed differences have been reported within these loci when other equid species were analyzed [5], [7], [14], despite sample sizes of other species being generally very low. Since the regions were already available in GenBank, some previous studies did not deposit additional domestic horse sequences, perhaps because no polymorphisms were found and by assuming that the already deposited sequences were identical to the additional sequences generated.

However, the sequences identified in all of our stallions in this study and in [7] were identical to one another but differed at 15 unique positions from the previously deposited sequences in GenBank. The disagreements included 13 single nucleotide differences and two indels. This level of divergence is highly unexpected, and is on the order of that observed between divergent equid species [5], [7], [14].

A couple of observations lead us to suspect that the sequence variation observed between studies springs from technical issues rather than true allele calls, i) sampling of the same breeds between studies but surprisingly different allele calls, and ii) differences between studies in how sequence data were obtained (see below). Two of the breeds sequenced for this study, the Spanish Purebred and the Lipizzan, were also included in previous studies [5]. Both previous larger studies using these markers included the Thoroughbred [5], [7] and the Thoroughbred sequenced by [7] had the same sequence observed in the Spanish Purebred, Lipizzan and all other breeds in [5]. It seems, therefore, highly unlikely that all individuals from these three breeds processed in one lab would actually have a different sequence from all stallions of the same breeds processed in a different lab. This is especially unexpected for the Thoroughbred, which has a very well recorded breeding history involving a limited number of sires.

In the course of analyzing our data we were able to compare our data to an alignment of the sequences generated by Wallner [22] which included four of the six loci containing putative differences, *Eca-Y2B17*, *Eca-Y3B1*, *Eca-Y3B12* and *Eca-Y3B19*. These four fragments include nine of the putative mutations. These alignments contained both vector cloned sequences and sequences directly obtained from amplification of horse DNA without a cloning step. Interestingly, only the cloned sequences match the sequences in GenBank. Because sequences from clones have a considerably higher error rate due to mis-incorporation of nucleotides by the *Taq* polymerase used (10–100 times higher) [26] than sequences generated directly from a PCR from genomic DNA, it is common practice to accept sequences from clones only after they have been encountered from multiple clones. The sequences in the alignment labeled horse (as opposed to clone) did not match the clone sequences, and did match the sequence we obtained at these four fragments. Therefore, it seems likely that the nine unverified sequence variants were based on single clones rather than the sequences generated by direct sequencing of genomic DNA, and it was the clone sequences which were submitted to GenBank (Table 3).

The reference sequences for locus *AMELY6* comes from a comparatives study in which this fragment of the Y chromosome was sequenced for several mammalian species [19]. There were three differences between our sequence and the reference in this 470 base pair fragment: two base pair changes and one indel (Table 3, Table S1). This study generated sequences for this fragment from several species, some from direct sequencing of PCR products, and some through the sequencing of clones from the PCR products. It looks like all templates were sequenced twice in both directions to ensure there were not sequencing errors. However, polymerase errors are very much more frequent and thus problematic than sequencing errors [26], [27]. It is possible that the horse sequence was based on the clone sequences, and this is how the three differences entered the dataset.

The final locus in which differences were identified between our sequence and the reference sequence is the 452 base pair fragment of the *SRY* gene (Table 3, Table S1). Three differences were identified in this fragment, two base pair differences and one indel. In the original publication [20], cDNA was amplified from testicular RNA and then cloned for sequencing. It is not mentioned in the publication if more than one clone was sequenced, and so *Taq* amplification errors exposed through sequencing of an insufficient number of clones could also explain these differences. In addition to this sequence, the same fragment from horse has also been deposited into GenBank two other times, although not associated with publications (AC215855 and HM103387). These sequences matched each other and our sequences, which suggests that these sequences more accurately reflect the genomic sequence of this fragment in the domestic horse. Taken altogether, these observations call into question the veracity of all 15 putative SNPs and indels.

### Microsatellite Variation

The six Y-chromosome microsatellite loci utilized here have also been employed in other studies involving over 1000 stallions (Table 5). Allele calling for microsatellite loci is not directly comparable between studies because different individuals could call the same allele differently, and the same allele can migrate in the polymer used to measure amplicons differently on different machines. For this reason the different sizes reported for the same locus in the different studies does not necessarily indicate allelic variation within the species. One study [6] reports three alleles for locus *Eca.YH12* (Table 5), however those three peaks form a consistent genotype that was found in the same form in all individuals, and thus was not variable in their sample [6]. Only one study found two variants for domestic horses within its dataset [15]. These two alleles at locus *Eca.YA16* identified in some local Chinese breeds is the only indication of standing Y-chromosome variation in the domestic horse.

### Domestication and Husbandry of Horses

Genetic and archaeological evidence suggests that horse domestication occurred more recently than the other primary Eurasian domesticates, perhaps around 6000 years ago [1], [28]–[31]. However, this is not known with certainty because domesticated and wild horses are very difficult to differentiate in the archaeological record [32] and the maternal and paternal genetic stories are apparently different, with high levels of haplotype diversity in the maternally-inherited mitochondrial DNA and almost no variation in the paternally inherited Y-chromosome [1]–[8], [14], [28]–[30], [33]–[36]. This could suggest recurrent backcrossing between limited numbers of stallions and numerous captured wild female horses around the time of domestication.

Our results support previous observations of near complete monomorphism on the Y-chromosome in extant domestic horses. This could be explained in various ways: 1) the genetic diversity on the Y-chromosome was already limited in the wild ancestral species before the domestication; or 2) genetic variation was lost in the bottleneck events associated with domestication; or 3) genetic variation was lost after domestication through genetic drift, selective breeding (i.e. breed formation and the “popular sire” effect); or 4) strong natural selection (selective sweep) after domestication. Hypothesis 1 could be supported by several factors that can explain the low levels of diversity of the Y-chromosome found in other wild mammals, such as sexual selection, mating system or sex-biased migration patterns, or other mechanisms that promote a small male effective population size [37], [38]. However, hypothesis 1 is undermined by the recent finding of abundant genetic diversity in the Y-chromosome sequences of ancient horses [14]. Hypothesis 2 is supported by some theories regarding domestication in which a strongly limited number of individuals are removed from the wild and closely controlled by humans. Hypothesis 3 could be supported by the selective breeding system that is still used today in horse breeds, in which “popular sires” reduce the effective male population size and thus decrease Y-chromosome diversity through genetic drift. For example, it is well documented that all modern Thoroughbred horses can trace back their pedigrees to three stallions imported to England from the Middle East in the late 17th and early 18th centuries. Domestication results in strong artificial selection for specific traits. It is possible that the severely reduced variation on Y-linked loci is a result of a combination of multiple factors, and further studies on the genetic variation of early domestic horses are necessary to critically evaluate their relative importance.

### Conclusion

In conclusion, the 15 SNPs in six of the 31 widely studied Y-chromosome specific fragments which we identified by comparing sequences generated in this study from 46 stallions originating from 12 breeds to sequences from other studies available in GenBank, likely all represent errors in the database. Our data support the previously observed absence of sequence variation and near-absence of microsatellite variation in extant domestic horse Y-chromosomes. We suggest that the unconfirmed sequences, GenBank accession AB091794 for *AMELY6*, AB004572 for *SRY*, G72335 for *Eca-Y2B17*, G72336 for *Eca-Y3B1*, G72338 for *Eca-Y3B12*, and G72339 for *Eca-Y3B19,* should no longer be used as reference sequences.

## Supporting Information

Table S1 Alignment of the six Y-chromosome specific loci.
(PDF)
