# Supplementary material for: Y-Chromosome Analysis in Retuertas Horses
Source: PLoS One. 2013 May 31;8(5):e64985. doi: 10.1371/journal.pone.0064985 (PMC3669082; doi:10.1371/journal.pone.0064985)
Supplement: Table S1 — Alignment of the six Y-chromosome specific loci. (PDF) [file pone.0064985.s001.pdf]

Table S1. Alignment of six Y chromosome specific loci identified as possibly polymorphic. Alignment includes the reference domestic horse sequence from GenBank, the sequences generated for this study (labeled TS), the sequences generated for [S1] and other equid sequences, as available. Each line is labeled first with the reference, then an abbreviation for the species as follows: Ec, *Equus caballus*; Ef, *E. ferus* (extinct wild progenitor species of domestic horse); Ep, *E. przwalskii*; Ea, *E. asinus*; Ek, *E. kiang*; Eba, *E. burchellii antiquorum*; Ebb, *E. burchellii boehmi*; Eg, *E. greyvi*; Ez, *E. zebra*. An ancient domestic horse (2,800 years old) from [S2] is marked with an asterisk.

a. Alignment of Y chromosome specific locus *Eca-Y2B17*. Reference sequence is GenBank accession G72335.

```
                   10        20        30        40        50        60        70        80        90       100
          ....|....|....|....|....|....|....|....|....|....|....|....|....|....|....|....|....|....|....|....|
[ref]Ec   TTCAGTCCTGCTTTCTCCTCATGGGCAGCTCTTTGATGTGACTGAGTCCTGCCTTTCCCTCTCAGGGGTGCTCCAGGCCCCGTCATGGAGAGGCAGGCAG
[TS]Ec    ---------------------...............................................................................
[S1]Ec    -----------------------------------.................................................................
[S2]Ec*   ----------------------------------------............................................................
[S2]Ef    ----------------------------------------............................................................
[S2]Ef_2  ----------------------------------------............................................................
[S2]Ef_3  ----------------------------------------............................................................
[S2]Ef_4  ----------------------------------------............................................................
[S2]Ef_5  ----------------------------------------............................................................
[S3]Ep    --------------------................................................................................
[S1]Ep    -----------------------------------.................................................................
[S3]Ea    --------------------................................................................................
[S1]Ea    -----------------------------------.................................................................
[S3]Ek    --------------------................................................................................
[S3]Eba   --------------------................................................................................
[S3]Ebb   --------------------................................................................................
[S3]Eg    --------------------..................................................A.............................
[S3]Ez    --------------------................................................................................

                  110       120       130       140       150       160       170       180       190       200
          ....|....|....|....|....|....|....|....|....|....|....|....|....|....|....|....|....|....|....|....|
[ref]Ec   CCCCATGGCTTCAGGCCTGTCAGCTGTGTGGCGGGATACTGGGTGGTACCTGGAG-----CCATGTCAGGATGCCAATTGGTCCCCCGCAGAAACAGGGC
[TS]Ec    .......................................................-----.................C......................
[S1]Ec    .......................................................-----.................C......................
[S2]Ec*   .......................................................-----.................C......................
[S2]Ef    .......................................................-----.................C......................
[S2]Ef_2  .......................................................-----.................C......................
[S2]Ef_3  .......................................................-----.................C........T.T...........
[S2]Ef_4  .......................................................-----.................C......................
[S2]Ef_5  .......................................................-----.................C......................
[S3]Ep    .......................................................-----.................C......................
[S1]Ep    .......................................................-----.................C......................
[S3]Ea    .......................................................TGGTA.................C......................
[S1]Ea    .......................................................TGGTA.................C......................
[S3]Ek    .......................................................TGGTA.................C.........-............
[S3]Eba   .......................................................TGGTA.................C......................
[S3]Ebb   .......................................................TGGTA.................C......................
[S3]Eg    .......................................................TGGTA.................C......................
[S3]Ez    .......................................................TGGTA.................C......................

                  210       220       230       240       250       260       270       280       290       300
          ....|....|....|....|....|....|....|....|....|....|....|....|....|....|....|....|....|....|....|....|
[ref]Ec   CCTAGTGGCCCCCTGAAGACATTGTCTTGTGGGCACACAGCAGTAGTGGCTCTGGCCACCACTGTCCCTTTTCCGGTCGGCAGCGCTGGGCCACCTGACC
[TS]Ec    ....................................................................................................
[S1]Ec    ....................................................................................................
[S2]Ec*   ........................................G...........................................................
[S2]Ef    ........................................G...........................................................
[S2]Ef_2  ........................................G...........................................................
[S2]Ef_3  .........T..............................G...........................................................
[S2]Ef_4  ........................................G...........................................................
[S2]Ef_5  ........................................G.................................A.........................
[S3]Ep    ........................................G...........................................................
[S1]Ep    ........................................G...........................................................
[S3]Ea    ...........................C............G................................T..........................
[S1]Ea    ...........................C............G................................T..........................
[S3]Ek    ...........................C............G................................T..........................
[S3]Eba   ...........................C............G................................T..........................
[S3]Ebb   ...........................C............G................................T..........................
[S3]Eg    ...........................C............G................................T..........................
[S3]Ez    ............G..............C............G................................T..........................

                  310       320       330       340       350       360       370       380       390       400
          ....|....|....|....|....|....|....|....|....|....|....|....|....|....|....|....|....|....|....|....|
[ref]Ec   TGAGTGGGGTTGTTGTCCTGGAAGCCCCCAGGGAGGGCCGCACGGAGCTGCTCCCTGGGCGACATGGCAGCACTAGGTCCCTCAGGTAGGAAGGGACTCT
[TS]Ec    ....................................................................................................
[S1]Ec    ....................................................................................................
[S2]Ec*   ....................................................................................................
[S2]Ef    ....................................................................................................
[S2]Ef_2  ....................................................................................................
[S2]Ef_3  ....................................................................................................
[S2]Ef_4  ....................................................................................................
[S2]Ef_5  ....................................................................................................
[S3]Ep    ....................................................................................................
[S1]Ep    ....................................................................................................
[S3]Ea    ........................-...........................................................................
[S1]Ea    ........................-...........................................................................
```

```
[S3]Ek      ........................-...........................................................................
[S3]Eba     ........................-...........................................................................
[S3]Ebb     ........................-...........................................................................
[S3]Eg      ........................-...........................................................................
[S3]Ez      ........................-...........................................................................

                     410       420       430       440       450       460       470       480       490       500
            ....|....|....|....|....|....|....|....|....|....|....|....|....|....|....|....|....|....|....|....|
[ref]Ec     CGTCACTGCACTGGCTCAGGTGCTGGCATCTCTGGGAAGGCTGAGGGCAACGCACATCCGTGCTAGACAGAGAGGCCTGAGGCTGGCTGGGGGCCTCGTG
[TS]Ec      ................................................................G...................................
[S1]Ec      ................................................................G........---------------------------
[S2]Ec*     ................................................................G.....................--------------
[S2]Ef      ................................................................G.....................--------------
[S2]Ef_2    T...............................................................G.....................--------------
[S2]Ef_3    ................................................................G.....................--------------
[S2]Ef_4    T...............................................................G.....................--------------
[S2]Ef_5    T...............................................................G.....................--------------
[S3]Ep      ................................................................G.............................------
[S1]Ep      ................................................................G........---------------------------
[S3]Ea      ..........................A.....................................G........................C....------
[S1]Ea      ..........................A.....................................G........---------------------------
[S3]Ek      ..................................................T.............G.............................------
[S3]Eba     ................................................................G.............................------
[S3]Ebb     ................................................................G.............................------
[S3]Eg      ................................................................G.............................------
[S3]Ez      ................................................................G.............................------

                     510       520       530
            ....|....|....|....|....|....|...
[ref]Ec     ATCAGAGGCCAGCCAATCACATGGCACATCCTG
[TS]Ec      .................................
[S1]Ec      ---------------------------------
[S2]Ec*     ---------------------------------
[S2]Ef      ---------------------------------
[S2]Ef_2    ---------------------------------
[S2]Ef_3    ---------------------------------
[S2]Ef_4    ---------------------------------
[S2]Ef_5    ---------------------------------
[S3]Ep      ---------------------------------
[S1]Ep      ---------------------------------
[S3]Ea      ---------------------------------
[S1]Ea      ---------------------------------
[S3]Ek      ---------------------------------
[S3]Eba     ---------------------------------
[S3]Ebb     ---------------------------------
[S3]Eg      ---------------------------------
[S3]Ez      ---------------------------------
```

b. Alignment of Y chromosome specific locus *Eca-Y3B1.* Reference sequence is GenBank accession G72336.

```
                  10        20        30        40        50        60        70        80        90       100
         ....|....|....|....|....|....|....|....|....|....|....|....|....|....|....|....|....|....|....|....|
[ref]Ec  TGGGTTAATGGGATTTGGTGGCTCCTCCTCCACCTGGAGAATGCAGCAGTGTAGACTTCTGGGAATTGAGGTCAGCATTGGGGAAAACTTAGCTCCTTAG
[TS]Ec   --------------------................................................................................
[S1]Ec   --------------------................................................................................
[S2]Ec*  ----------------------------------------------------------..........................................
[S2]Ef   ----------------------------------------------------------..................C.......................
[S2]Ef_2 ----------------------------------------------------------..................C.......................
[S2]Ef_3 ----------------------------------------------------------..................C.......................
[S2]Ef_4 ----------------------------------------------------------..........................................
[S2]Ef_5 ----------------------------------------------------------..........................................
[S2]Ef_6 ----------------------------------------------------------..........................................
[S3]Ep   --------------------------------------..............................................................
[S1]Ep   --------------------------------------------------------------------------------......................
[S3]Ea   --------------------------------------.......................................C..............T.......
[S1]Ea   --------------------......G.........A........................................C..............T.......
[S3]Ek   --------------------------------------...................................G...C..............T.......
[S3]Eba  --------------------------------------.......................................C..............T.......
[S3]Ebb  --------------------------------------.......................................C..............T.......
[S3]Eg   --------------------------------------.......................................C..............T...G...
[S3]Eh   --------------------------------------.......................................C..............T.......
[S3]Ez   --------------------------------------.......................................C..............T.......

                 110       120       130       140       150       160       170       180       190       200
         ....|....|....|....|....|....|....|....|....|....|....|....|....|....|....|....|....|....|....|....|
[ref]Ec  TAGACAAAGCTGCAGGTATTGAGTATTAGGAGCAAGGAAGGAAAGACTCTGGGGAACCATGTATCATGGCAGCAGCTCTCTCTCTCTATTGGGGCAGC
[TS]Ec   ....................................................................................................
[S1]Ec   ....................................................................................................
[S2]Ec*  ....................................................................................................
[S2]Ef   ....................................................................................................
[S2]Ef_2 ....................................................................................................
[S2]Ef_3 ....................................................................................................
[S2]Ef_4 ....................................................................................................
[S2]Ef_5 ....................................................................................................
[S2]Ef_6 ..........................................................................................A.........
[S3]Ep   ....................................................................................................
[S1]Ep   ....................................................................................................
[S3]Ea   .....................................................C...................................G..........
[S1]Ea   .....................................................C...................................G..........
[S3]Ek   .....................................................C...................................G..........
[S3]Eba  .....................................................C.........................T.........G..........
[S3]Ebb  .....................................................C.........................T.........G..........
[S3]Eg   .....................................................C...................................G..........
[S3]Eh   .....................................................C...................................G..........
[S3]Ez   .....................................................C...................................G..........

                 210       220       230       240       250       260       270       280       290       300
         ....|....|....|....|....|....|....|....|....|....|....|....|....|....|....|....|....|....|....|....|
[ref]Ec  GGCTGTGGCTTCAGCCTGGCTGAGACTGGCGGGTGACAACATCTCCTTCACCAGTGGGAGTGCCCGCAGCACCTTCTTTCTGAATTCCACAGGTGCTGTG
[TS]Ec   A...............................................................A...................................
[S1]Ec   A...............................................................A...................................
[S2]Ec*  A...............................................................A...................................
[S2]Ef   A.............................T.................................A.T.................................
[S2]Ef_2 A.............................T.................................A...................................
[S2]Ef_3 A.............................T.................................A...................................
[S2]Ef_4 A...............................................................A...................................
[S2]Ef_5 A...A...........................................................A...................................
[S2]Ef_6 A...............................................................A...................................
[S3]Ep   A...............................................................A...................................
[S1]Ep   A...............................................................A...................................
[S3]Ea   A...C........................T..................................A...................................
[S1]Ea   A...C........................T..................................A...................................
[S3]Ek   A...C........................T..................................A...................................
[S3]Eba  A...C........................T..................................A...................................
[S3]Ebb  A...C........................T..................................A...................................
[S3]Eg   A...C........................T....A.............................A........................T..........
[S3]Eh   A...C........................T..................................A...................................
[S3]Ez   A...C........................T..................................A...................................

                 310       320       330       340       350       360       370       380       390       400
         ....|....|....|....|....|....|....|....|....|....|....|....|....|....|....|....|....|....|....|....|
[ref]Ec  GCTCCCTCTCCAGGAGAAGTGCAATGTGGATTCCAGGCAGCTTCTTCAGCTGGGCTTGGCCTTGGTAAAGACTGCAAGAGTCCTCAGTGACAAAAGTTGC
[TS]Ec   ....................................................................................................
[S1]Ec   ....................................................................................................
[S2]Ec*  ....................................................................................................
[S2]Ef   ....................................................................................................
[S2]Ef_2 ....................................................................................................
[S2]Ef_3 ....................................................................................................
[S2]Ef_4 ....................................................................................................
[S2]Ef_5 ..........................................................A.T.......................................
[S2]Ef_6 ....................................................................................................
[S3]Ep   ....................................................................................................
[S1]Ep   ....................................................................................................
[S3]Ea   ....................................................................................................
[S1]Ea   ....................................................................................................
[S3]Ek   ....................................................................................................
[S3]Eba  ....................................................................................................
[S3]Ebb  ....................................................................................................
[S3]Eg   ....................................................................................................
[S3]Eh   ....................................................................................................
```

```
[S3]Ez     ....................................................................................................

                   410       420       430       440       450       460       470       480       490       500
           ....|....|....|....|....|....|....|....|....|....|....|....|....|....|....|....|....|....|....|....|
[ref]Ec    AGTTGTCCAAAGCAGTGTCAGGGCCTGCTGAGTTCCTCTTATTTTCTTTTTCCCTCATGACGAGAGATTGCAACTGAAGGGATCTCTCTTGATACAGAGC
[TS]Ec     ....................A...............................................................................
[S1]Ec     ....................A...................................................................------------
[S2]Ec*    ....................A...............................................--------------------------------
[S2]Ef     ....................A...............................................--------------------------------
[S2]Ef_2   ....................A...............................................--------------------------------
[S2]Ef_3   ....................A...............................................--------------------------------
[S2]Ef_4   ....................A...............................................--------------------------------
[S2]Ef_5   ....................A...............................................--------------------------------
[S2]Ef_6   ....................A...............................................--------------------------------
[S3]Ep     ....................A................................-----------------------------------------------
[S1]Ep     ....................A...................................................................------------
[S3]Ea     ......G.............A................................-----------------------------------------------
[S1]Ea     ......G.............A..............................................C....................------------
[S3]Ek     ......G.............A................................-----------------------------------------------
[S3]Eba    ....................A................................-----------------------------------------------
[S3]Ebb    ....................A................................-----------------------------------------------
[S3]Eg     ....................A................................-----------------------------------------------
[S3]Eh     ......G.............A................................-----------------------------------------------
[S3]Ez     ....................A................................-----------------------------------------------


           ....|...
[ref]Ec    TGTGCTTG
[TS]Ec     ........
[S1]Ec     --------
[S2]Ec*    --------
[S2]Ef     --------
[S2]Ef_2   --------
[S2]Ef_3   --------
[S2]Ef_4   --------
[S2]Ef_5   --------
[S2]Ef_6   --------
[S3]Ep     --------
[S1]Ep     --------
[S3]Ea     --------
[S1]Ea     --------
[S3]Ek     --------
[S3]Eba    --------
[S3]Ebb    --------
[S3]Eg     --------
[S3]Eh     --------
[S3]Ez     --------
```

c. Alignment of Y chromosome specific locus *Eca-Y3B12.* Reference sequence is GenBank accession G72338.

```
                    10        20        30        40        50        60        70        80        90       100
            ....|....|....|....|....|....|....|....|....|....|....|....|....|....|....|....|....|....|....|....|
[ref]Ec     GGGAGGCACTGGAAAGTACACTCTTCTGCAGGCCTGCTACCCCAGGTCGCTGCCCCTGGTGACTCCCAGTCCCTGTCTACACCATGAGTGCAGAGGTTGT
[TS]Ec      ----------------------------------------........A...................................................
[S1]Ec      ----------------------------------------........A...................................................
[S2]Ec*     -------------------------------------------------...................................................
[S2]Ef      -------------------------------------------------.....................T.............................
[S2]Ef_2    -------------------------------------------------...................................................
[S2]Ef_3    -------------------------------------------------.........................................T.........
[S3]Ep      --------------------------------....................................................................
[S1]Ep      ----------------------------------------........A...................................................
[S3]Ea      --------------------------------................A.......................T...........................
[S1]Ea      --------------------....................A.......................T...........................
[S3]Eba     --------------------------------................A.......................T...........................
[S3]Ebb     --------------------------------................A.......................T...........................
[S3]Eg      --------------------------------................A.......................T...........................
[S3]Eh      --------------------------------................A.......................T...........................
[S3]Ez      ---------------------------------...............A.......................T...........................

                   110       120       130       140       150       160       170       180       190       200
            ....|....|....|....|....|....|....|....|....|....|....|....|....|....|....|....|....|....|....|....|
[ref]Ec     GTTCTTCCTCGCCAAGGCCTGGCAGTTTTCACACCTAAGAGTCCACATCCTTGGCTGCCTCAAGGCAGGCAGCAAGCCTTCTCTCAGCCTTAAAAACATG
[TS]Ec      ....................................................................................................
[S1]Ec      ....................................................................................................
[S2]Ec*     ....................................................................................................
[S2]Ef      ....................................................................................................
[S2]Ef_2    ....................................................................................................
[S2]Ef_3    ....................................................................................................
[S3]Ep      ....................................................................................................
[S1]Ep      ....................................................................................................
[S3]Ea      ................................................A...................................................
[S1]Ea      ................................................A...................................................
[S3]Eba     ................................................A...................................................
[S3]Ebb     ................................................A...................................................
[S3]Eg      ................................................A...................................................
[S3]Eh      ................................................A.................................A.................
[S3]Ez      ................................................A...................................................

                   210       220       230       240       250       260       270       280       290       300
            ....|....|....|....|....|....|....|....|....|....|....|....|....|....|....|....|....|....|....|....|
[ref]Ec     CCTAGAATTTTGCAAGAGAAGTTGGAAATGGCAACTGCTCCAGAAATAGCCTTTGGTTTGGGCTTATTTGCACAAGTAGGCGTGGTGGGGTGACCCTGAC
[TS]Ec      ................................................................................T...................
[S1]Ec      ................................................................................T...................
[S2]Ec*     ................................................................................T...................
[S2]Ef      ................................................................................T...................
[S2]Ef_2    ................................................................................T...................
[S2]Ef_3    ................................................................................T...................
[S3]Ep      ....................................................................................................
[S1]Ep      ................................................................................T...................
[S3]Ea      ................................................................................T.C.................
[S1]Ea      ................................................................................T.C.................
[S3]Eba     ................................................................................T.C.................
[S3]Ebb     ................................................................................T.C.................
[S3]Eg      ................................................................................T.C...........T.....
[S3]Eh      ..............................................................T.................T.C.................
[S3]Ez      ................................................................................T.C.................

                   310       320       330       340       350       360       370       380       390       400
            ....|....|....|....|....|....|....|....|....|....|....|....|....|....|....|....|....|....|....|....|
[ref]Ec     AGAACCAGCGCTTCCAAAAGCTTCCTCTGGCCAGGGCTTCCCTGCCCATTAATAAATACCAGTGTAGTGTAAAGAGTCAGCTCCAGCTGATTCCTCCACC
[TS]Ec      ....................................................................................................
[S1]Ec      ................................................................................--------------------
[S2]Ec*     ................................................................................--------------------
[S2]Ef      ................................................................................--------------------
[S2]Ef_2    ................................................................................--------------------
[S2]Ef_3    ................................................................................--------------------
[S3]Ep      ................................................................................--------------------
[S1]Ep      ................................................................................--------------------
[S3]Ea      .....T..T.......................................................................--------------------
[S1]Ea      .....T..T.......................................................................--------------------
[S3]Eba     .....T...............................................................C..........--------------------
[S3]Ebb     .....T...............................................................C..........--------------------
[S3]Eg      .....T..........................................................................--------------------
[S3]Eh      .....T..T.......................................................................--------------------
[S3]Ez      .....T..........................................................................--------------------
```

d. Alignment of Y chromosome specific locus *Eca-Y3B19.* Reference sequence is GenBank accession G72339.

```
                   10        20        30        40        50        60        70        80        90       100
          ....|....|....|....|....|....|....|....|....|....|....|....|....|....|....|....|....|....|....|....|
[ref]Ec   AAGCCTTTCATGGAAATTGGCTTGATCCAAACTGCTCCTTTAGCAGCCGAAACCGTAAAACATAAAGCAATAAATCCAATAAAAAAGAAAGAAAGAAACT
[TS]Ec    --------------------................................................................................
[S1]Ec    --------------------................................................................................
[S2]Ec*   ....................................................................................................
[S2]Ef    .....................................................T.....G........................................
[S2]Ef_2  .....................................................T.....G........................................
[S2]Ef_3  .....................................................T..............................................
[S2]Ef_4  .....................................................T..............................................
[S3]Ep    --------------------.................................T..............................................
[S1]Ep    --------------------.................................T..............................................
[S3]Ea    --------------------........................C........T..............................................
[S1]Ea    --------------------........................C........T..............................................
[S3]Ek    --------------------........................C........T..............................................
[S3]Eba   --------------------........................C........T..............................................
[S3]Ebb   --------------------........................C........T..............................................
[S3]Eg    --------------------........................C........T..............................................
[S3]Eh    --------------------........................C........T..............................................

                  110       120       130       140       150       160       170       180       190       200
          ....|....|....|....|....|....|....|....|....|....|....|....|....|....|....|....|....|....|....|....|
[ref]Ec   CATTCTCCGCCGGATTGGGCTGCTCTGTTTTGTTTTGTTTTTTGTTGTTTCAAGTATAGTGCAGCATCTTCTCAGGAGAGTGACTTTGAGGGCAGGGGAG
[TS]Ec    ..............A..........................................................................A..........
[S1]Ec    ..............A..........................................................................A..........
[S2]Ec*   ..............A..........................................................................A..........
[S2]Ef    ..............A...........................................................A..............A..........
[S2]Ef_2  ..............A...........................................................A..............A..........
[S2]Ef_3  ..............A..........................................................................A..........
[S2]Ef_4  ..............A..........................................................................A..........
[S3]Ep    ..............A..........................................................................A..........
[S1]Ep    ..............A..........................................................................A..........
[S3]Ea    ..............A...........................................A..............................A..........
[S1]Ea    ..............A...........................................A..............................A..........
[S3]Ek    ..............A...........................................A..............................A......A...
[S3]Eba   ..............A...........................................A..............................A..........
[S3]Ebb   ..............A...........................................A..............................A..........
[S3]Eg    ..............A...........................................A..............................A..........
[S3]Eh    ..............A...........................................A..............................A..........

                  210       220       230       240       250
          ....|....|....|....|....|....|....|....|....|....|....|
[ref]Ec   GGCGGTTTTAAAACCAGGTGGCTGGCGAGGAGCCCTGTCCAGGATGTCTGCGTAA
[TS]Ec    .......................................................
[S1]Ec    ...................................--------------------
[S2]Ec*   ...................................--------------------
[S2]Ef    ...................................--------------------
[S2]Ef_2  ...................................--------------------
[S2]Ef_3  ...................................--------------------
[S2]Ef_4  ...................................--------------------
[S3]Ep    ...................................--------------------
[S1]Ep    ...................................--------------------
[S3]Ea    .............T.....................--------------------
[S1]Ea    .............T.....................--------------------
[S3]Ek    ...................................--------------------
[S3]Eba   ...................................--------------------
[S3]Ebb   ...................................--------------------
[S3]Eg    ...................................--------------------
[S3]Eh    ...................................--------------------
```

e. Alignment of Y chromosome specific locus *Amely6.* Reference domestic horse sequence is GenBank accession AB091794.

```
                  10        20        30        40        50        60        70        80        90       100
          ....|....|....|....|....|....|....|....|....|....|....|....|....|....|....|....|....|....|....|....|
[ref]Ec   AAACTCTTTTATGAAATACGAGCATTAAAGAAAAATAAATTCTTAATTAATTTGACATCTAAGGGAGATATTCTGCACGATTCTCTATTCTTTCTCTCTC
[TS]Ec    -------------------------...........................................................................
[S1]Ec    ....................................................................................................
[S2]Ec*   ........................................................................................C...........
[S2]Ef    ........................................................................................C...........
[S2]Ef_2  ........................................................................................C...........
[S2]Ef_3  ........................................................................................C...........
[S2]Ef_4  ........................................................................................C...........
[S2]Ef_5  ........................................................................................C...........
[S1]Ep    ........................................................................................C...........
[S1]Ea    ..............................................................................C.........C...........

                 110       120       130       140       150       160       170       180       190       200
          ....|....|....|....|....|....|....|....|....|....|....|....|....|....|....|....|....|....|....|....|
[ref]Ec   ACCCACTCCAGCACCAATACAAATTGCACAACACAGGCATCGGTCATTCGGCCTCCACATTTGAAAAGACAGTATTGGTTTAGATAATTATTCAGTTTGC
[TS]Ec    ....................................................................................................
[S1]Ec    ....................................................................................................
[S2]Ec*   ....................................................................................................
[S2]Ef    ....................................................................................................
[S2]Ef_2  ....................................................................................................
[S2]Ef_3  ....................................................................................................
[S2]Ef_4  ....................................................................................................
[S2]Ef_5  ....................................................................................................
[S1]Ep    ....................................................................................................
[S1]Ea    .............................................................................A............C.....G...

                 210       220       230       240       250       260       270       280       290       300
          ....|....|....|....|....|....|....|....|....|....|....|....|....|....|....|....|....|....|....|....|
[ref]Ec   TTTTCACTTTGACCTTTAACATTTTTCTGCCGTAAAAACATTGACAGGGTAATAAAAGTTGGCCTAAACTTCATAAATCAACTTCTATTTTTCATAGGAT
[TS]Ec    ...............................A....................................................................
[S1]Ec    ...............................A....................................................................
[S2]Ec*   ...............................A....................................................................
[S2]Ef    ...............................A....................................................................
[S2]Ef_2  ...............................A....................................................................
[S2]Ef_3  ...............................A....................................................................
[S2]Ef_4  ...............................A....................................................................
[S2]Ef_5  ...............................A....................................................................
[S1]Ep    ...............................A....................................................................
[S1]Ea    .............................T.A....................................................................

                 310       320       330       340       350       360       370       380       390       400
          ....|....|....|....|....|....|....|....|....|....|....|....|....|....|....|....|....|....|....|....|
[ref]Ec   TTATAATGTTTCTACTTTACCCATCTTGCTCTCACATACATCAATAATGCCCAAAATAATTGTTTCTTTTGTTGTTGTTGATGTGTTATTTACTTTGCAT
[TS]Ec    ............................T.......................-...............................................
[S1]Ec    ............................T.......................-...............................................
[S2]Ec*   ....................................................................................................
[S2]Ef    ............................T.......................................................................
[S2]Ef_2  ............................T.......................................................................
[S2]Ef_3  ............................T.......................................................................
[S2]Ef_4  ............................T.......................................................................
[S2]Ef_5  ....................................................................................................
[S1]Ep    ............................T.......................-...............................................
[S1]Ea    ............................T.............G.........-...C...........................................

                 410       420       430       440       450       460       470       480
          ....|....|....|....|....|....|....|....|....|....|....|....|....|....|....|....|....|
[ref]Ec   GCCACTTCTGAGGACTAGTTAAGTATTTGGTAGTTAAGAATAGTTAACTGCCTTTGATAGTTAAGTATTTATCCATGTTGTGATC
[TS]Ec    .....................................................................................
[S1]Ec    .......................................................................--------------
[S2]Ec*   .....................................................................................
[S2]Ef    .....................................................................................
[S2]Ef_2  .....................................................................................
[S2]Ef_3  .....................................................................................
[S2]Ef_4  .....................................................................................
[S2]Ef_5  .....................................................................................
[S1]Ep    .......................................................................--------------
[S1]Ea    ..................C...............................T....................--------------
```

f. Alignment of Y chromosome specific locus *SRY.* Reference sequence is GenBank accession AB004572.

```
                     10        20        30        40        50        60        70        80        90       100
            ....|....|....|....|....|....|....|....|....|....|....|....|....|....|....|....|....|....|....|....|
[ref]Ec     CCTCCGGACTTTCTCACGGTGATTTTCCTTGATTTCCTTACTGGTGGCTTCAGAGTCCCTGTTCGGCTCTATTTTCGTATTTCTTCTGGAGCCCCAGCGT
[TS]Ec      ------------------------........................GG..................................................
[S1]Ec      ------------------------........................GG..................................................
AC215855Ec  ----............................................GG..................................................
HM103387Ec  ................................................GG..................................................
[S2]Ec*     --..............................................GG..................................................
[S2]Ef      --..............................................GG..................................................
[S1]Ep      ------------------------........................GG..................................................
[S1]Ea      ------------------------........................GG.........................A..............A......A..
EU240939Ea  ................................................GG.........................A..............A......A..
EU240940Eb  ................................................GG.........................A..............A......A..
EU240941Eg  ................................................GG.........................A..............A......A..
EU240942Ez  ................................................GG.........................A..............A......A..

                    110       120       130       140       150       160       170       180       190       200
            ....|....|....|....|....|....|....|....|....|....|....|....|....|....|....|....|....|....|....|....|
[ref]Ec     ATCAACACCAGTGAAATTTTGAGTTCCAAGGTCAGCTGTTTTTCTGTTAATGGAACAGTTTGTAATCTAATTTTAGTCTTCCAGAGGTTGTCCTTTAAAT
[TS]Ec      ....................................................................................................
[S1]Ec      ....................................................................................................
AC215855Ec  ....................................................................................................
HM103387Ec  ....................................................................................................
[S2]Ec*     ....................................................................................................
[S2]Ef      ....................................................................................................
[S1]Ep      ....................................................................................................
[S1]Ea      ....................................................................................................
EU240939Ea  ....................................................................................................
EU240940Eb  ....................................................................................................
EU240941Eg  ....................................................................................................
EU240942Ez  ....................................................................................................

                    210       220       230       240       250       260       270       280       290       300
            ....|....|....|....|....|....|....|....|....|....|....|....|....|....|....|....|....|....|....|....|
[ref]Ec     AGCGCTAAGCATATATTGATACACTAATAATCGCCAGCATAGATCACAGAATTCTAACTCTTTATTTTAAATACTATAAGTCACAAACATAGTGGAGAGA
[TS]Ec      ....................................................................................................
[S1]Ec      ....................................................................................................
AC215855Ec  ....................................................................................................
HM103387Ec  ....................................................................................................
[S2]Ec*     ....................................................................................................
[S2]Ef      ....................................................................................................
[S1]Ep      ....................................................................................................
[S1]Ea      .......................................................................................T............
EU240939Ea  .......................................................................................T............
EU240940Eb  .......................................................................................T............
EU240941Eg  .......................................................--..............................T............
EU240942Ez  .......................................................................................T............

                    310       320       330       340       350       360       370       380       390       400
            ....|....|....|....|....|....|....|....|....|....|....|....|....|....|....|....|....|....|....|....|
[ref]Ec     AGCATGCACAAAATTATGCTATGGAAAAATTGTTGCGTTTTGATGGGAAACTGCGTA-CGTCTACAGTAGTCCCTTCTCGACGGTCTGTTCATTGGTCTT
[TS]Ec      .........................................................G..........................................
[S1]Ec      .........................................................G..........................................
AC215855Ec  .........................................................G..........................................
HM103387Ec  .........................................................G..........................................
[S2]Ec*     .........................................................G..........................................
[S2]Ef      .........................................................G..........................................
[S1]Ep      .........................................................G..........................................
[S1]Ea      .........................................................G.........A................................
EU240939Ea  .........-------------------------------------------------------------------------------------------
EU240940Eb  .........-------------------------------------------------------------------------------------------
EU240941Eg  .........-------------------------------------------------------------------------------------------
EU240942Ez  .........-------------------------------------------------------------------------------------------

                    410       420       430       440       450       460       470       480       490       500
            ....|....|....|....|....|....|....|....|....|....|....|....|....|....|....|....|....|....|....|....|
[ref]Ec     TCAGCTACCTTGCGCGTCTTGGTAAACTGCACATAATTAACGCATGGAAATGTAAATTCTTTCCTGTTGGATAGTTTATGTTACCTCCCCTCCCCGCTGT
[TS]Ec      ....................................................................................................
[S1]Ec      .........................................................................---------------------------
AC215855Ec  ....................................................................................................
HM103387Ec  ....................................................................................................
[S2]Ec*     ............................................................................------------------------
[S2]Ef      ............................................................................------------------------
[S1]Ep      ............................................................................------------------------
[S1]Ea      ............................................................................------------------------
EU240939Ea  ----------------------------------------------------------------------------------------------------
EU240940Eb  ----------------------------------------------------------------------------------------------------
EU240941Eg  ----------------------------------------------------------------------------------------------------
EU240942Ez  ----------------------------------------------------------------------------------------------------
```

```
                     510
            ....|....|....|.
[ref]Ec     GAGAAACCAGTCTTGC
[TS]Ec      ...............-
[S1]Ec      ----------------
AC215855Ec  ................
HM103387Ec  ................
[S2]Ec*     ----------------
[S2]Ef      ----------------
[S1]Ep      ----------------
[S1]Ea      ----------------
EU240939Ea  ----------------
EU240940Eb  ----------------
EU240941Eg  ----------------
EU240942Ez  ----------------
```

References

S1. Lindgren G, Backström N, Swinburne J, Hellborg L, Einarsson A, Sandberg K, Cothran G, Vila C, Binns M, Ellegren H (2004) Limited number of patrilines in horse domestication. Nat Genet 36: 335-336. DOI:10.1038/ng1326

S2. Lippold S, Knapp M, Kuznetsova T, Leonard JA, Benecke N, Ludwig A, Rasmussen M, Weinstock J, Willerslev E, Shapiro B, Hofreiter M (2011) Discovery of lost diversity of paternal horse lineages using ancient DNA. Nat Commun 2:450. DOI: 10.1038/ncomms1447.

S3. Wallner B, Brem G, Müller M, Achmann R (2003) Fixed nucleotide differences on the Y-chromosome indicate clear divergence between Equus przewalskii and Equus caballus. Anim Genet 34: 453-456. DOI: 10.1046/j.0268-9146.2003.01044.x
